# Study design of herbal medicine clinical trials: a descriptive analysis of published studies investigating the effects of herbal medicinal products on human participants

**DOI:** 10.1186/s12906-024-04697-7

**Published:** 2024-11-08

**Authors:** Nut Koonrungsesomboon, Chotiwit Sakuludomkan, Mingkwan Na Takuathung, Preeyaporn Klinjan, Suphunwadee Sawong, Pathirage Kamal Perera

**Affiliations:** 1 Department of Pharmacology, Faculty of Medicine, Chiang Mai University, Chiang Mai, 50200 Thailand; 2 Clinical Research Center for Food and Herbal Product Trials and Development (CR-FAH), Faculty of Medicine, Chiang Mai University, Chiang Mai, 50200 Thailand; 3 Department of Ayurveda Pharmacology, Pharmaceutics, and Community Medicine, Faculty of Indigenous Medicine, University of Colombo, Rajagiriya, Sri Lanka

**Keywords:** Clinical trials, Herbal medicines, Study design

## Abstract

**Background:**

Increasing global interest in natural therapies has led to a rise in the use of herbal medicines for managing various ailments. However, concerns about scientific evaluation have prompted a study aiming to assess the study design of herbal medicine clinical trials. This study aimed to provide a descriptive overview of the study design, characteristics, and methodologies of contemporary herbal medicine clinical trials.

**Materials and methods:**

The study reviewed herbal medicine clinical trials published between 2019 and 2022 in five electronic databases: PubMed, Embase, Web of Sciences, Scopus, and the Cochrane Library. Data extraction included study characteristics, intervention details, study design, outcome measures, trial phases, blinding, and other relevant information, with descriptive analyses presented. The term ‘herbal medicines’ in this study refers to herbs, herbal materials, preparations, and finished products containing active ingredients from plant parts or their combinations.

**Results:**

Out of the initially identified 5,918 records, 1,517 articles were eligible for inclusion in the study. The majority of herbal medicine clinical trials were conducted in Asian countries, covering a range of diseases. A randomized, double-blind, parallel design with a 1:1 allocation ratio was frequently employed, along with the common use of placebos across all trial phases. Capsules were the most common dosage form. The median number of human participants varied across trial phases, ranging from 50 in Phase 1 to 240 in Phase 4.

**Conclusions:**

The analysis observed that herbal medicine clinical trials employed randomized, double-blind, parallel designs, and the widespread use of placebo. Our observations provided valuable insights into the evolving landscape of herbal medicine clinical trials.

**Supplementary Information:**

The online version contains supplementary material available at 10.1186/s12906-024-04697-7.

## Background

The use of herbal medicines continues to expand rapidly around the world, with an increasing number of people turning to them for the maintenance of good health and for the treatment and/or prevention of physical and mental illnesses [1]. Over the last decade, natural therapies have gained greater acceptance and public interest, with herbal medicines readily available in drugstores and grocery stores in both developing and developed countries [2, 3]. Many herbs have been known to contain bioactive compounds with pharmacological properties, making them a promising source for drug discovery and as a complementary and alternative therapy [4, 5]. In the current era, researchers are keenly interested in exploring the therapeutical properties of herbal medicinal plants to unlock their full potential for further development [6, 7]. Despite the growing global use of herbal medicines and the introduction of new herbal medicinal products to the market, many of them have not undergone scientific and clinical evaluation, nor has their use in humans been systematically monitored [8]. This lack of scientific evidence for herbal medicine therapy raises concerns about its efficacy and safety, limiting its acceptance in the current era of evidence-based medicine.

Clinical trials are considered the gold standard for assessing the efficacy and safety of therapeutics, and as such, an increasing number of studies have been conducted to evaluate the scientific evidence of herbal medicinal products [9]. However, there are distinct characteristics of herbal medicine clinical trials that differ from those of conventional chemical drugs or other therapeutics. There are some methodological difficulties and ethical issues specifically related to the study design of herbal medicine clinical trials [10, 11]. For instance, the gold standard randomized-controlled trial may not apply to the holistic and/or individualistic approaches in traditional medicine, where some herbal medicines may need to be customized for individual patients [12]. Additionally, preparing a placebo control of an herbal medicinal product with a distinctive smell and/or color may be challenging [13, 14]. As trial design can significantly impact the validity and reliability of trial results, an inappropriate design may hinder the acceptance of herbal medicines as effective and safe outside of their native cultural contexts [15].

Therefore, the objective of the present study was to provide a descriptive overview of the study design, characteristics, and methodologies currently used in herbal medicine clinical trials.

## Methods

### Study protocol, search strategy, and selection criteria

The study protocol for review was developed to determine the study designs of herbal medicine clinical trials of published papers. It was granted an exemption by the Research Ethics Committee of the Faculty of Medicine, Chiang Mai University (No. EXEMPTION-08849/2022). A systematic search was conducted in July 2021 (last update on December 31, 2022) across five electronic databases, namely PubMed, Embase, Web of Sciences, Scopus, and the Cochrane Library, using terms related to ‘herbal medicine’ and ‘clinical trials.’ The search was limited to original research articles published between 2019 and 2022, with no language restrictions.

After the initial search, the identified records were imported into EndNote X9, and duplicates were removed. Articles were screened based on title and abstract, and those deemed potentially relevant were retrieved for further assessment. Full-text articles were considered for inclusion if they reported clinical trials that used herbal medicine as an intervention and involved human participants, regardless of age, gender, or health condition. The term ‘herbal medicines’ in this study includes herbs, herbal materials, herbal preparations, and finished herbal products, all of which contain active ingredients derived from parts of plants, or other plant materials, or combinations thereof. These products could be in various forms, such as extracts, powders, or capsules, provided they retained the natural composition of the plant material. Importantly, products in which the active substances had undergone chemical modifications, additions, or synthesis, such as the inclusion of synthetic compounds or isolated constituents, were excluded from the definition of herbal medicine in this study.

Articles that did not present original clinical data were excluded, including review articles, case reports, expert opinions, conference abstracts, comments, editorials, and surveys. Additionally, studies that did not involve human participants, such as animal or in vitro studies, were not included. Furthermore, clinical trials that used combination treatments, where herbal medicine was not the primary intervention, were excluded to maintain focus on those evaluating the efficacy of herbal medicines. To ensure comprehensiveness, the reference lists of all relevant articles were screened for additional potentially eligible studies.

Two independent reviewers (C.S., P.K., and/or S.S.) conducted the screening and selection of studies to ensure a rigorous and unbiased process. Initially, the reviewers screened the titles and abstracts of all identified studies based on predefined inclusion and exclusion criteria. Any discrepancies between the reviewers were resolved through discussion, and a third reviewer (N.K. and/or M.N.) was consulted in cases where consensus could not be reached. After the initial screening, full-text articles of potentially eligible studies were retrieved and further assessed for inclusion. Any disagreements were resolved by discussion or by involving a third reviewer.

### Data extraction

Data were extracted from the included studies with the following information: (1) study characteristics (i.e., sponsor/funder, study location, and number of human participants), (2) intervention characteristics (i.e., type of the herbal medicine intervention, comparator, route of administration, and intervention duration), (3) study design (i.e., trial phase, trial category, trial type, trial framework, randomization, blinding, number of arms, and allocation ratio), and (4) type of outcome measures (i.e., subjective (soft) outcomes (e.g., pain perception) or objective (hard) outcomes (e.g., measurable biomarkers)). Trial registration and protocol availability were also determined based on publication details. The health outcomes were classified according to the International Classification of Diseases (ICD) codes of diagnosis categories, blocks of categories, and/or chapters according to the ICD-10 WHO version 2019.

For this study, the trial phase was categorized into four phases based on the glossary of common site terms used on ClinicalTrials.gov, i.e., Phase 1, Phase 2, Phase 3, and Phase 4. In addition, clinical trials were categorized as either explanatory (designed to test the efficacy or safety of an intervention in an ideal context) or pragmatic (aimed at evaluating its effectiveness and safety in real-world practice). The clinical trial designs were classified into different trial types, i.e., (1) parallel design, where each participant is randomly assigned to receive either the intervention or control in separate groups; (2) crossover design, where each participant receives both the intervention and control but in a different order; (3) factorial design, which is suited for the study of two or more interventions in various combinations in one study setting; and (4) cluster design, where a clinical trial involves randomization by study sites or locations rather than by individual participants. Moreover, the framework of a clinical trial was also extracted, if available, based on the study’s hypothesis: superiority, non-inferiority, or equivalence.

For this study, the blinding terminology is categorized as follows: (1) “double-blind”, indicating a clinical trial in which both participants and investigators/outcome assessors were blinded; (2) “single-blind”, indicating a clinical trial in which either participants or investigators/outcome assessors were blinded; and (3) “open-label”, indicating a clinical trial in which neither participants nor investigators/outcome assessors were blinded.

### Data analysis

Descriptive analyses were presented as either frequency with percentage (%) or mean with standard deviation, depending on data type. The statistical analyses and visualization were performed using RStudio software version 4.0.3.

## Results

**Fig. 1:**
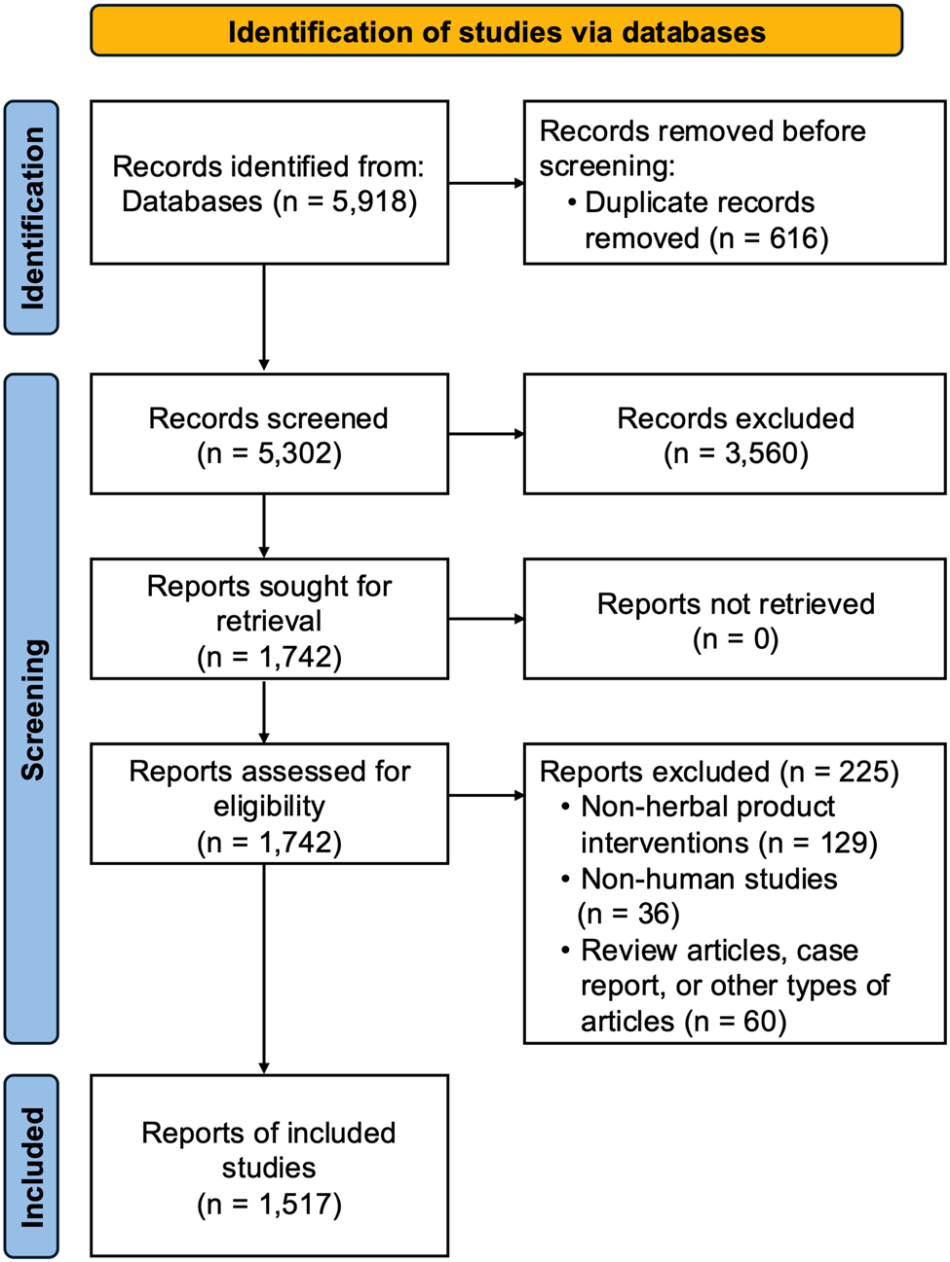
Flow chart diagram

Of 5,918 records identified through the initial database searches, 5,302 were screened, and 1,742 were retrieved for full-text reading and assessed for eligibility. Finally, a total of 1,517 articles were found to be eligible for inclusion, with the reasons for exclusion outlined in Fig. 1. More than two-thirds of the included studies (*n* = 984, 64.9%) were single-center studies, while nearly a quarter of the studies (*n* = 346, 22.8%) were conducted in multiple centers. About three-fourths of the publications (*n* = 1,114, 73.4%) declared funding sources, two-thirds of which (*n* = 1,012, 66.7%) were governmental funds. Herbal medicine clinical trials were conducted in various countries, with the majority in Asian countries (*n* = 1,272, 83.8%). The diseases for which the herbal intervention was tested were categorized based on the ICD-10 diagnosis codes (Supplementary Table S1). The majority of these fell into the following groups: diseases of endocrine, nutritional, and metabolic diseases (*n* = 166, 10.9%), diseases of the digestive system (*n* = 160, 10.5%), genitourinary system (*n* = 135, 8.9%), healthy volunteer (*n* = 130, 8.6%), and diseases of the circulatory system (*n* = 126, 8.3%).

Nearly two-thirds of the included studies (*n* = 967, 63.7%) investigated a complex mixture of multiple herbs in an herbal formulation, while nearly one-third (*n* = 459, 30.3%) examined a single herb extract. Only a few of them (*n* = 91, 6.0%) investigated a standardized herbal formulation in humans. Capsule was the most common dosage form of herbal medicinal products (*n* = 434, 28.6%), followed by granule (*n* = 259, 17.1%), tablet (*n* = 137, 9.0%), and decoction (*n* = 102, 6.7%).

**Fig. 2:**
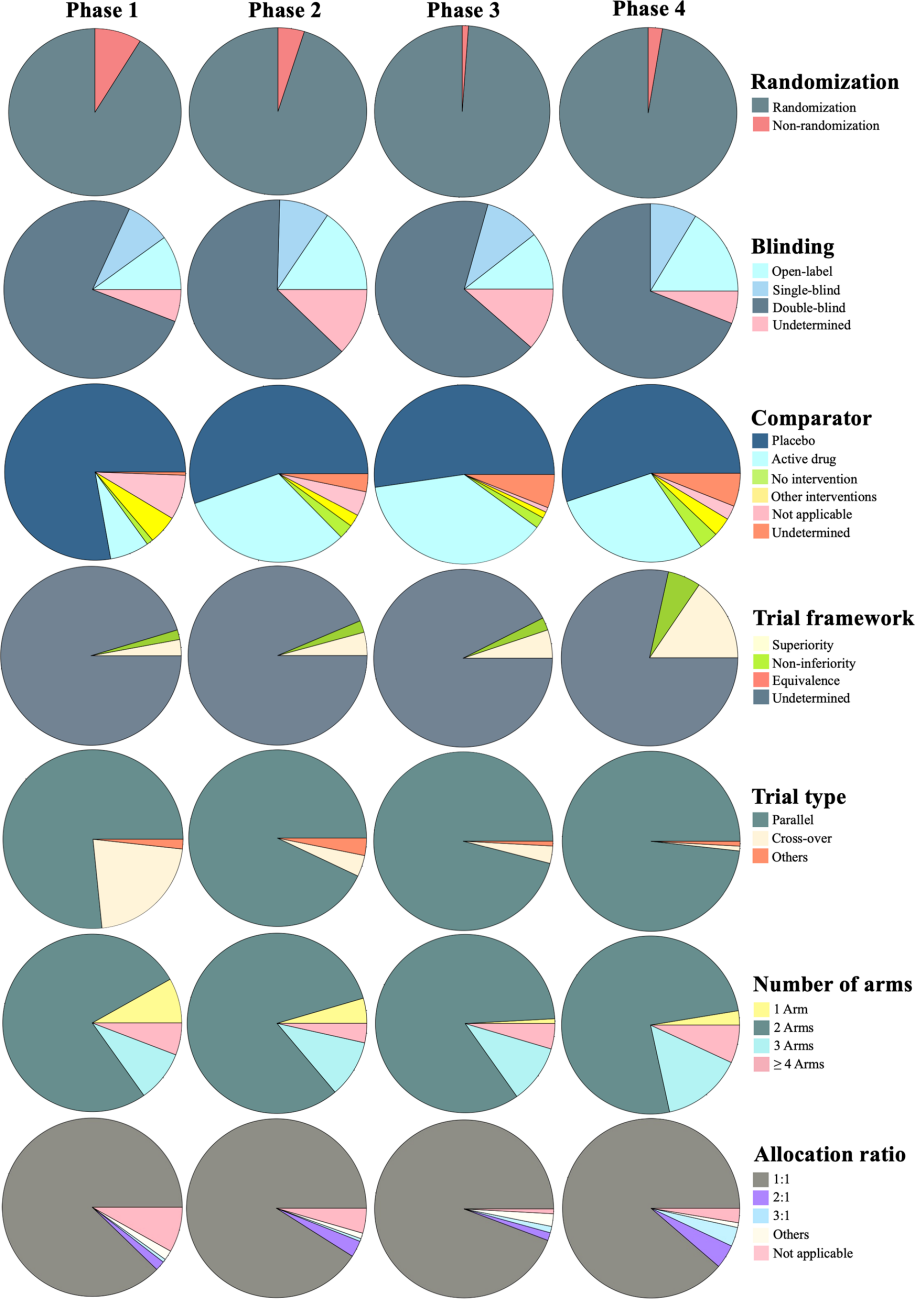
The distribution of study designs in each clinical trial phase

The study design used in the included studies is summarized in Table 1; Fig. 2. The majority of studies employed a randomized (*n* = 1452, 95.7%), double-blind (*n* = 993, 65.5%), parallel design (*n* = 1398, 92.2%), with a 1:1 allocation ratio (*n* = 1382, 91.1%). Randomization was a commonly employed technique across all trial phases, including Phase 1, with a usage rate of up to 91.2%. The double-blind technique was the most frequently used in herbal medicine clinical trials across all trial phases, with usage rates of 76%, 63.3%, 67.8%, and 69% in Phase 1, Phase 2, Phase 3, and Phase 4, respectively. Meanwhile, single-blind and open-label techniques were also observed across all trial phases of herbal medicine clinical trials.

**Table 1:** Characteristics of the included studies

Variables	Number of the included studies (%)
**Trial phase**	
1	171 (11.3%)
2	977 (64.4%)
3	348 (22.9%)
4	116 (7.6%)
Undetermined	1 (0.1%)
**Trial category**	
Explanatory	1403 (92.5%)
Pragmatic	114 (7.5%)
**Randomization**	
Yes	1452 (95.7%)
No	4 (0.3%)
Not applicable (for a single-arm study)	61 (4.0%)
**Blinding**	
Open-label	214 (14.1%)
Single-blind	136 (9.0%)
Double-blind	993 (65.5%)
Undetermined	174 (11.5%)
**Comparator**	
Placebo	856 (56.4%)
Active drug	465 (30.7%)
No intervention	39 (2.6%)
Another intervention	39 (2.6%)
Not applicable (for a single-arm study)	61 (4.0%)
Undetermined	57 (3.8%)
**Trial framework**	
Superiority	79 (5.2%)
Non-inferiority	37 (2.4%)
Equivalence	0 (0.0%)
Undetermined	1401 (92.4%)
**Trial type**	
Parallel	1398 (92.2%)
Crossover	81 (5.3%)
Factorial	3 (0.2%)
Undetermined	35 (2.3%)
**Number of arms**	
1 arm	61 (4.0%)
2 arms	1228 (80.9%)
3 arms	163 (10.7%)
≥ 4 arms	65 (4.3%)
**Allocation ratio**	
1:1	1382 (91.1%)
2:1	40 (2.6%)
3:1	12 (0.8%)
Others	5 (0.3%)
Not applicable (for a single-arm study)	61 (4.0%)
Undetermined	17 (1.1%)

In over half of the included studies across all trial phases, a placebo was commonly used (77.8% in Phase 1, 55.5% in Phase 2, 52.3% in Phase 3, and 55.2% in Phase 4). An active drug comparator was sometimes used in clinical trial phases 2–4 (31.8% in Phase 2, 37.6% in Phase 3, and 29.3% in Phase 4).

A parallel design was commonly used across all trial phases (76.6% in Phase 1, 92.9% in Phase 2, 96.0% in Phase 3, and 98.3% in Phase 4), while a crossover design was sometimes seen in Phase 1 clinical trials (21.6%). Nearly all of the included studies (*n* = 1,401, 92.4%) did not explicitly indicate the trial framework whether they employed a superiority, non-inferiority, or equivalence design.

Two arms were most used across all trial phases (79.2% in Phase 1, 82.9% in Phase 2, 86.0% in Phase 3, and 75.0% in Phase 4), while a single-arm design was sometimes used in Phase 1 (8.2%) and Phase 2 (4.5%) clinical trials and was seldom or rarely used in Phase 3 (0.9%) or Phase 4 (2.6%) clinical trials. For clinical trials with more than one arm, an allocation ratio of 1:1 was mostly employed, with 87.7% in Phase 1, 91.0% in Phase 2, 94.3% in Phase 3, and 88.8% in Phase 4, while unequal allocation (such as 2:1 or 3:1) could be seen in herbal medicine clinical trials across all trial phases, albeit rarely.

The majority of the included studies (*n* = 1,001, 66.0%) measured both subjective and objective outcomes, while some only measured objective outcomes (*n* = 394, 26.0%). A few studies only had subjective outcome measures (*n* = 122, 8.0%).

**Fig. 3:**
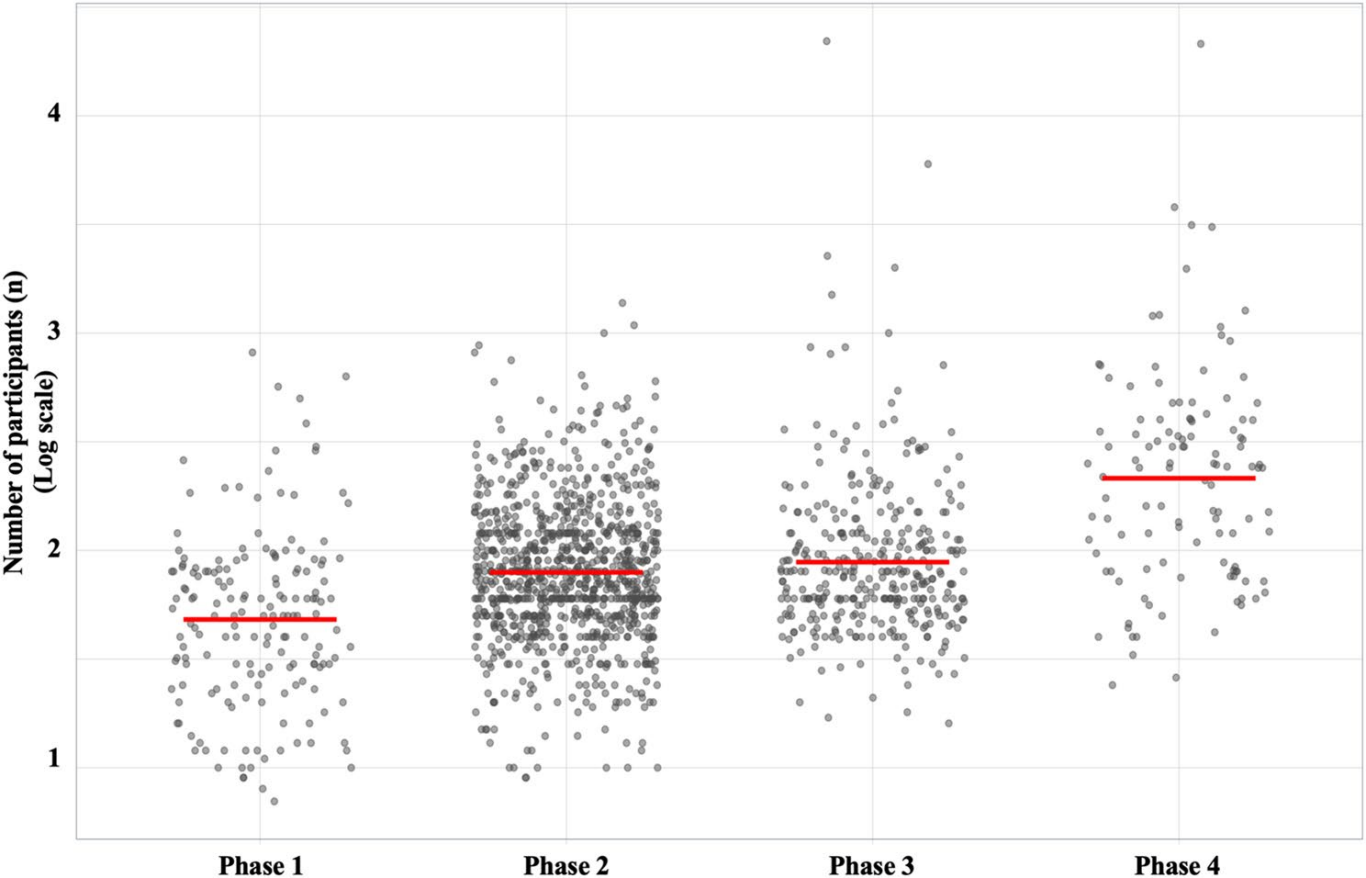
The number of human participants enrolled in clinical trial phases

The number of human participants in clinical trials varied by trial phases, with a median of 50.0 (IQR, 27.0–82.0) for Phase 1, 76.0 (IQR, 50.0–120.0) for Phase 2, 76.0 (IQR, 54.0–120.0) for Phase 3, and 240.0 (IQR, 84.0–400.0) for Phase 4 (Fig. 3).

## Discussion

The present study provides a descriptive analysis of the study design of herbal medicine clinical trials based on a review of original research articles published between 2019 and 2022. The majority of these clinical trials were single-center, predominantly conducted in Asian countries, addressing a diverse range of diseases. Our study observed various interesting issues that merit further discussion as follows.

First, our analysis revealed that a substantial proportion of studies compared herbal medicinal products with placebos to assess their efficacy and/or safety. In placebo-controlled clinical trials, it is crucial that the placebo closely resembles the investigational product in terms of visual attributes, dosage form, smell, and taste to maintain the scientific validity of the trial. The distinctive characteristics of herbal product preparations present challenges in placebo preparation compared to chemical compound placebos [16]. It is important to highlight that achieving a perfectly matched control substance, especially for herbal product preparations with complex constituents and varied dosage forms such as decoctions, can be challenging or even impractical. The present study underscores that capsules were the most common dosage form for herbal medicinal products, and experts have noted their relative simplicity in achieving the physical resemblance of placebos compared to other forms [17]. Nevertheless, challenges remain, particularly for herbal medicinal products with unique constituents and forms. For instance, decoctions and other liquid preparations present additional difficulties in creating indistinguishable placebos, potentially impacting the validity of placebo-controlled clinical trials [18]. Therefore, the creation of effective placebos for dosage forms other than capsules, particularly decoctions, remains a significant challenge. Future research should explore innovative approaches to placebo-controlled design that can address these challenges and ensure the robustness of placebo-controlled clinical trials in herbal medicine.

Second, while a double-blind design was the most frequently employed method across all trial phases, approximately a quarter of the included studies opted for either a single-blind or open-label design. Although double-blinding is the gold standard for minimizing bias and ensuring the robustness of clinical trials, certain situations necessitate the use of single-blind or open-label designs [19]. This is particularly true for herbal medicinal products with distinctive sensory characteristics, such as strong odors or vivid colors, which can inadvertently unblind participants and researchers [20]. Such design limitations can introduce biases and potentially compromise the validity and reliability of trial results [21].

Third, we observed a discrepancy between the sample sizes in herbal medicine clinical trials and those typically seen in conventional drug development clinical trials, particularly in Phase 3. Our review found that the median enrollment in Phase 3 clinical trials for herbal medicinal products was relatively small, which does not align with the large participant numbers commonly enrolled in confirmatory Phase 3 clinical trials in conventional drug development. In drug trials, Phase 3 studies often involve thousands of participants to confirm the efficacy and assess common adverse effects of the drugs. However, the smaller sample sizes observed in these herbal medicine clinical trials may be expected, as many of the studies were not conducted for regulatory purposes [22]. The sample size may have been determined based on the type of outcomes measured, rather than regulatory requirements. As such, the small sample size alone is not necessarily a significant finding without further exploration of how outcome types influence these decisions. Additionally, our review did not aim to provide an in-depth analysis of trial phases, such as the rationale for sample size or the specific characteristics of early-phase trials. For instance, Phase 2 clinical trials may focus on dose-ranging or proof-of-concept studies, affecting the number of participants and study design [23]. Future research could provide valuable insights by investigating these factors more thoroughly.

It should be noted that our study focused exclusively on original research articles, sourced from five major electronic databases (i.e., PubMed, Embase, Web of Science, Scopus, and the Cochrane Library). While these databases are widely recognized for their comprehensive coverage of clinical trials, some clinical trials on Eastern herbal medicine may not be published in journals indexed by these databases [24, 25]. This may overlook significant research conducted in regions where traditional and herbal medicine practices are more common, particularly in Asia [26]. Future studies should incorporate more region-specific databases or other repositories focused on traditional medicine to provide a more globally representative analysis.

Another limitation of this study is that it was not initially designed to gather detailed information on the comparators. Future studies would benefit from a comprehensive evaluation of comparators, including those related to herbal medicine, traditional therapies, and Western medicine. Such an investigation would provide valuable insights into the nature of the comparators and their potential influence on study outcomes. Furthermore, while the outcomes measured were relevant to the study’s objectives, a deeper examination of the different types of outcomes could have enhanced the interpretation of results. Exploring a broader range of outcome types in future research could offer a more understanding of their impact on the findings.

Last but not least, it should be noted that a risk of bias assessment of the included studies was not performed, as the primary objective of this review was to describe key study characteristics, such as study design, sample size, and types of interventions in herbal medicine clinical trials, rather than assessing intervention effects. Our focus was therefore on summarizing the methodological features of these trials rather than evaluating outcomes or efficacy. Given that this review did not involve synthesizing or comparing intervention effects, a formal risk of bias assessment was not deemed necessary [27]. However, we acknowledge that not evaluating the risk of bias may limit insights into the methodological rigor of the included studies. Incorporating a risk of bias assessment in future reviews could strengthen interpretation by providing greater interpretative depth, potentially highlighting areas where evidence is more robust or where caution may be warranted in interpreting results.

## Conclusions

In conclusion, our review of 1,517 eligible articles sheds light on the landscape of herbal medicine clinical trials. Notably, two-thirds of the clinical trials investigated complex mixtures of multiple herbs, with capsules being the most common dosage form. The prevailing study designs favored randomized, double-blind, parallel structures with a 1:1 allocation ratio. Placebos were commonly employed across all trial phases, and the majority of studies measured both subjective and objective outcomes. The number of human participants varied across trial phases, with the median ranging from 50 in Phase 1 to 240 in Phase 4. This comprehensive analysis provides valuable insights into the characteristics and methodologies of contemporary herbal medicine clinical trials.

## Electronic supplementary material

Below is the link to the electronic supplementary material.

Supplementary Material 1

## Data Availability

The datasets used and/or analyzed during the current study are available from the corresponding author upon reasonable request.

## Abbreviations

CONSORT: Consolidated Standards of Reporting Trials
ICD: International Classification of Diseases
IQR: Interquartile range
