# Supplementary material for: Study design of herbal medicine clinical trials: a descriptive analysis of published studies investigating the effects of herbal medicinal products on human participants
Source: BMC Complement Med Ther. 2024 Nov 8;24:391. doi: 10.1186/s12906-024-04697-7 (PMC11549860; doi:10.1186/s12906-024-04697-7)
Supplement: Supplementary file 1 — Supplementary Material 1 [file 12906_2024_4697_MOESM1_ESM.docx]

**Supplementary Table S1.** Conditions being studied (ICD10)

| **Code** | **Variables** | **Number of the included studies (%)** |
| --- | --- | --- |
| I | Certain infectious and parasitic diseases | 48 (3.2%) |
| II | Neoplasms | 106 (7.0%) |
| III | Diseases of the blood and blood-forming organs and certain disorders involving the immune mechanism | 6 (0.4%) |
| IV | Endocrine, nutritional, and metabolic diseases | 166 (10.9%) |
| V | Mental and behavioral disorders | 89 (5.9%) |
| VI | Diseases of the nervous system | 49 (3.2%) |
| VII | Diseases of the eye and adnexa | 6 (0.4%) |
| VIII | Diseases of the ear and mastoid process | 4 (0.3%) |
| IX | Diseases of the circulatory system | 126 (8.3%) |
| X | Diseases of the respiratory system | 98 (6.5%) |
| XI | Diseases of the digestive system | 160 (10.5%) |
| XII | Diseases of the skin and subcutaneous tissue | 74 (4.9%) |
| XIII | Diseases of the musculoskeletal system and connective tissue | 83 (5.5%) |
| XIV | Diseases of the genitourinary system | 135 (8.9%) |
| XV | Pregnancy, childbirth, and the puerperium | 7 (0.5%) |
| XVI | Certain conditions originating in the perinatal period | 5 (0.3%) |
| XVII | Congenital malformations, deformations, and chromosomal abnormalities | 0 (0.0%) |
| XVIII | Symptoms, signs, and abnormal clinical and laboratory findings, not elsewhere classified | 18 (1.2%) |
| XIX | Injury, poisoning, and certain other consequences of external causes | 11 (0.7%) |
| XX | External causes of morbidity and mortality | 4 (0.3%) |
| XXI | Factors influencing health status and contact with health services | 5 (0.3%) |
| XXII | Codes for special purposes | 57 (3.8%) |
|  | No underlying conditions (i.e., healthy volunteers) | 130 (8.6%) |
|  | Undetermined | 130 (8.6%) |
